# Lipid-Lowering and Anti-Inflammatory Effects of *Campomanesia adamantium* Leaves in Adipocytes and *Caenorhabditis elegans*

**DOI:** 10.3390/ph17081062

**Published:** 2024-08-13

**Authors:** Paola dos Santos da Rocha, Sarah Lam Orué, Isamara Carvalho Ferreira, Priscilla Pereira de Toledo Espindola, Maria Victória Benites Rodrigues, José Tarcísio Giffoni de Carvalho, Debora da Silva Baldivia, Daniel Ferreira Leite, Helder Freitas dos Santos, Alex Santos Oliveira, Jaqueline Ferreira Campos, Edson Lucas dos Santos, Kely de Picoli Souza

**Affiliations:** Research Group on Biotechnology and Bioprospecting Applied to Metabolism (GEBBAM), Federal University of Grande Dourados, Rodovia Dourados-Itahum, Km 12, Dourados 79804-970, MS, Brazil; paolasantosrocha@ufgd.edu.br (P.d.S.d.R.); sarah.orue005@academico.ufgd.edu.br (S.L.O.); isamara.ferreira137@academico.ufgd.edu.br (I.C.F.); priscatoledo@hotmail.com (P.P.d.T.E.); maria.rodrigues072@academico.ufgd.edu.br (M.V.B.R.); tarcisiogiffoni@outlook.com.br (J.T.G.d.C.); deborasbaldivia@ufgd.edu.br (D.d.S.B.); danielleitesci@gmail.com (D.F.L.); helderfsantos@ufgd.edu.br (H.F.d.S.); alexsantosoliveira@gmail.com (A.S.O.); jaquelinefcampos@ufgd.edu.br (J.F.C.); edsonsantosphd@gmail.com (E.L.d.S.)

**Keywords:** guavira, oxidative stress, inflammation, obesity

## Abstract

Obesity is a pandemic disease characterized by lipid accumulation, increased proinflammatory cytokines, and reactive oxygen species. It is associated with the development of comorbidities that lead to death. Additionally, drug treatments developed to control obesity are insufficient and have a variety of adverse effects. Thus, the search for new anti-obesity therapies is necessary. *Campomanesia adamantium* is a species from the Brazilian Cerrado that has the potential to treat obesity, as described by the antihyperlipidemic activity of its roots. Therefore, this study aimed to investigate the activity of the aqueous extract of *C. adamantium* leaves (AECa) on the control of reactive species in vitro, on lipid accumulation in adipocytes and *Caenorhabditis elegans*, and on the production of proinflammatory cytokines in adipocytes. The antioxidant capacity of AECa was observed by its action in scavenging DPPH^•^ free radical, iron-reducing power, and inhibition of β-carotene bleaching. AECa reduced lipid accumulation in preadipocytes and in *C. elegans*. Moreover, AECa reduced the production of the proinflammatory cytokines MCP-1, TNF-α, and IL-6 in adipocytes. In summary, the antioxidant activity and the ability of AECa to reduce the accumulation of lipids and proinflammatory cytokines indicate, for the first time, the anti-obesity potential of *C. adamantium* leaves.

## 1. Introduction

Obesity is a pandemic chronic inflammatory disease, mainly due to unhealthy eating and a sedentary lifestyle. It is currently the leading nutritional disorder in developed countries [1]. Obesity is characterized by the expansion of white adipose tissue, resulting from the processes of adipogenesis and lipogenesis, which are characteristics of adipocyte hyperplasia and hypertrophy, respectively [2]. Adipogenesis is upregulated in the mesenchymal stem cells of adipose tissue by fatty acid-activated transcription factors, which include PPAR-γ [3]. Furthermore, the activation of PPAR-γ in adipocytes also promotes the sensitivity of these cells to insulin, promoting the absorption of glucose and the accumulation of lipids through a process known as lipogenesis [4]. For lipid accumulation during lipogenesis, transcriptional factors, including SREBP-1c, are activated [5].

In addition, adipocyte hypertrophy contributes to the production of proinflammatory cytokines such as interleukin 6 (IL-6) and tumor necrosis factor-alpha (TNF-α) and increases the secretion of chemokines such as monocyte chemoattractant protein 1 (MCP-1) [6]. Hypertrophy upregulates a cycle of continuous cellular recruitment of inflammation that leads to the increased production of reactive oxygen species [7]. If not neutralized, these reactive oxygen species generate an oxidative stress condition. The inflammatory and oxidative stress conditions in adipocytes that characterize obesity are associated with the development of comorbidities such as type 2 diabetes, cardiovascular disease, and cancer, which are among the leading causes of death in the world today [8].

Drug treatments developed to control obesity are insufficient and have several adverse effects [9]. Therapeutic alternatives are, therefore, being sought to obtain products that modulate adipogenesis, lipogenesis, and the production of proinflammatory cytokines and possess antioxidant activities.

Among the therapeutic alternatives, medicinal plants have gained prominence for their biological activities, with fewer adverse effects and low costs to public health [10]. *Campomanesia adamantium* O. Berg, popularly known as guavira, is a medicinal plant from the Myrtaceae family, native to the Brazilian Cerrado [11]. *C. adamantium* is traditionally used to treat diarrhea, urinary tract infections, and stomach disorders [11]. Its biological properties include anti-inflammatory, pro-longevity, and cyclooxygenase-inhibiting activities [12,13,14].

In a previous study, we verified the antioxidant and antihyperlipidemic effects of *Campomanesia adamantium* O. Berg roots [15]. Furthermore, another study found that the aqueous extract of *C. adamantium* leaves has quinic acid and phenolic compounds, such as flavonoids, with myricetin and quercetin as its main constituents [16]. Therefore, in this study, we investigated the effect of *C. adamantium* leaves on controlling reactive oxygen species, lipid accumulation, and the production of proinflammatory cytokines.

## 2. Results

### 2.1. Antioxidant Activity of AECa

AECa showed antioxidant activity observed by the DPPH^•^, FRAP, and β-carotene bleaching methods (Table 1). The concentration of AECa capable of inhibiting 50% (IC_50_) of DPPH^•^ radicals was approximately 1.6 times higher than that of the Controls, ascorbic acid, and BHA. The FRAP test’s average effective concentration (EC_50_) of AECa was approximately 1.4 times higher than ascorbic acid. In the β-carotene bleaching test, AECa had an IC_50_ approximately 15 times higher than BHA, and a value the IC_50_ of ascorbic acid was not detected (Table 1).

### 2.2. Effect of AECa on Preadipocytes

**Figure 1:**
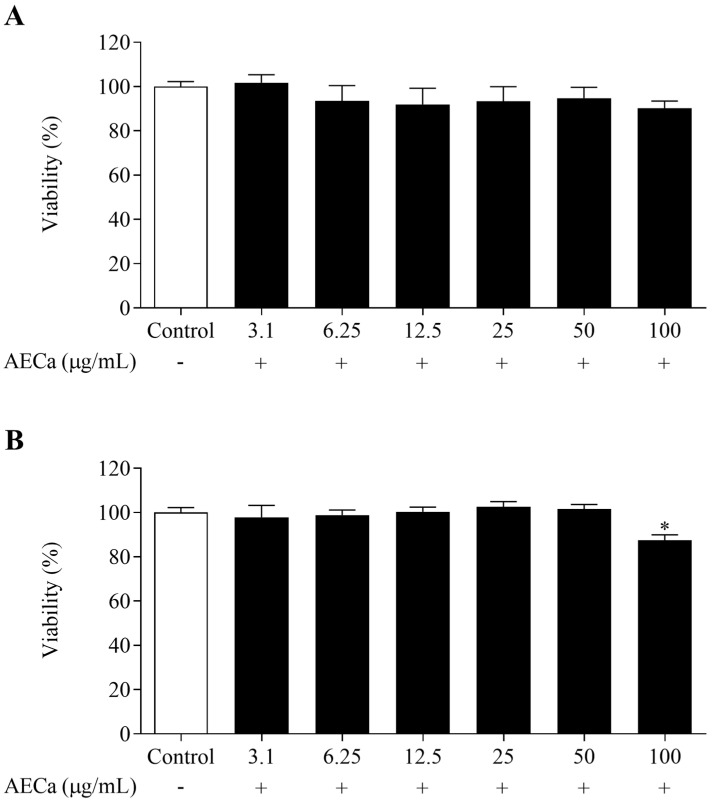
Cellular viability of preadipocytes incubated with different concentrations (3.1, 6.25, 12.5, 25, 50, and 100 µg/mL) of aqueous extract of *C. adamantium* leaves (AECa): (**A**) 24 h and (**B**) 48 h. Results are expressed as means ± SEM. * *p* < 0.05 versus Control.

AECa did not reduce the viability of preadipocytes regardless of the concentration at 24 h. At 48 h, the concentrations of AECa maintained viability above 90% (Figure 1).

### 2.3. Effect of AECa on Lipid Accumulation in Cells

**Figure 2:**
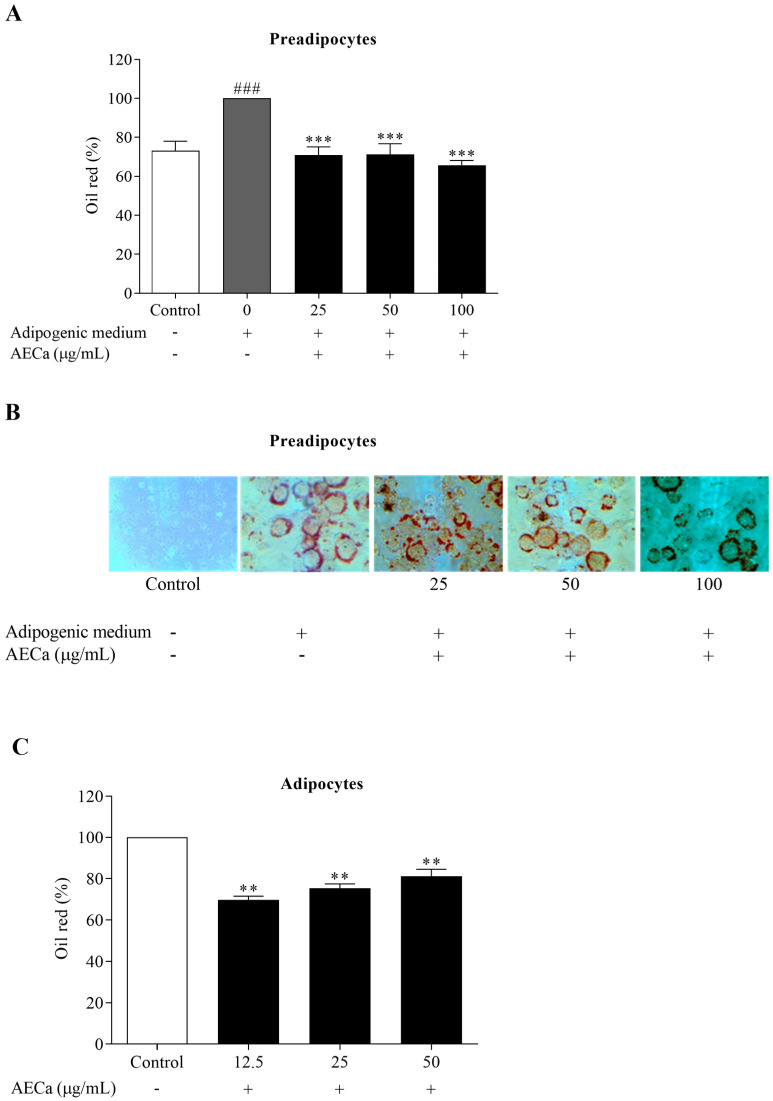
Lipid content observed by the percentage of Oil red in preadipocytes and differentiated adipocytes treated with different concentrations (0, 25, 50, and 100 µg/mL) of aqueous extract of *C. adamantium* leaves (AECa): (**A**) percentage of Oil red in preadipocytes treated with AECa followed by an 8-day induction of differentiation; (**B**) photomicrographs of preadipocytes treated with AECa followed by an 8-day induction of differentiation at 400× magnification; (**C**) percentage of Oil red in adipocytes by an 8-day differentiation induction followed by 48 h AECa treatment. Results are expressed as means ± SEM. ### *p* < 0.001 versus Control; ** *p* < 0.01, *** *p* < 0.01 versus adipogenic medium (0 µg/mL).

The induction of differentiation using adipogenic medium in preadipocytes increased lipid content by 35% compared to the lipid content of undifferentiated cells (Control). AECa prevented lipid accumulation in a concentration-independent manner by approximately 31% compared to the cells differentiated using adipogenic medium (Figure 2A,B).

In differentiated adipocytes, AECa reduced lipid accumulation by approximately 30%, 25%, and 19% at 12.5, 25, and 50 µg/mL concentrations, respectively, compared to the Control (Figure 2C).

### 2.4. Effect of AECa on Cytokine Production

**Figure 3:**
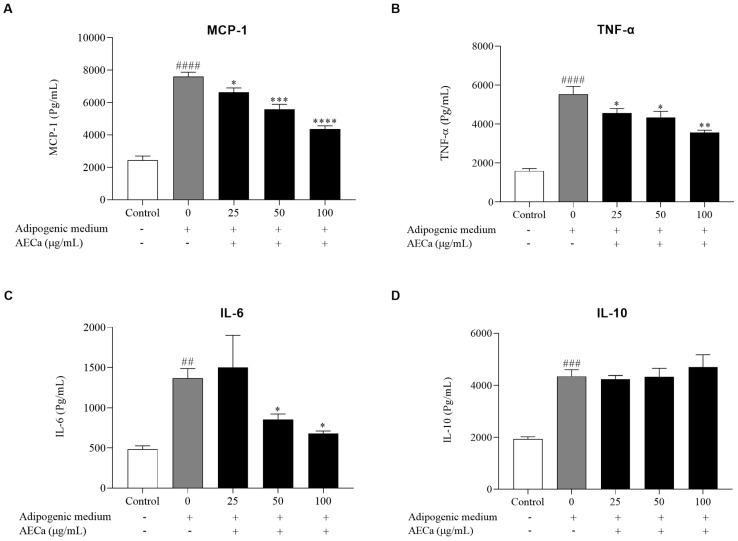
Cytokine production in preadipocytes and hypertrophied adipocytes treated with different concentrations (0, 25, 50, and 100 µg/mL) of aqueous extract of *C. adamantium* leaves (AECa): (**A**) MCP-1; (**B**) TNF-α; (**C**) IL-6; and (**D**) IL-10. Results are expressed as means ± SEM. ## *p* < 0.01, ### *p* < 0.001, and #### *p* < 0.0001 versus Control; * *p* < 0.05, ** *p* < 0.01, *** *p* < 0.001, and **** *p* < 0.0001 versus adipogenic medium.

The induction of differentiation by the adipogenic medium in preadipocytes increased the production of the proinflammatory cytokines MCP-1, TNF-α, and IL-6 compared to undifferentiated cells (Control). AECa reduced MCP-1 production by 13, 27, and 43% at 25, 50, and 100 µg/mL concentrations, respectively. AECa reduced TNF-α production by approximately 25%. AECa reduced IL-6 production by 37 and 50% at concentrations of 50 and 100 µg/mL. AECa did not alter the production of the anti-inflammatory cytokine IL-10 (Figure 3).

### 2.5. Effect of AECa on Caenorhabditis elegans Nematodes

**Figure 4:**
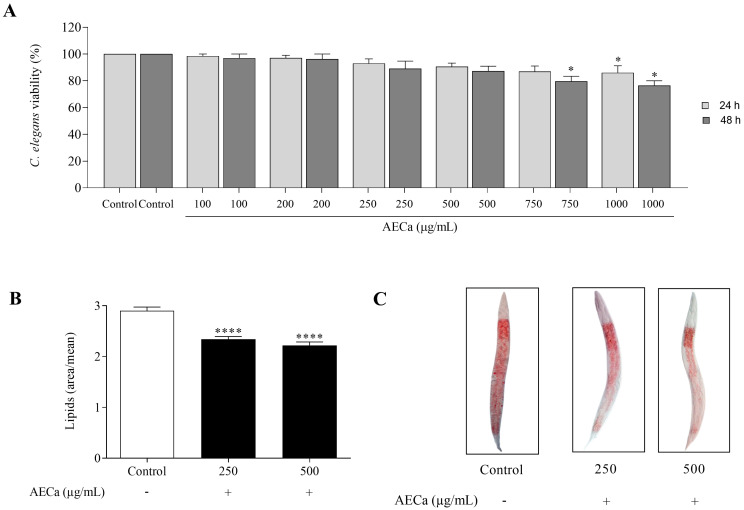
Viability and lipid content of *C. elegans* treated with different concentrations (100, 200, 250, 500, 750, and 1000 µg/mL) of aqueous extract of *C. adamantium* leaves (AECa): (**A**) 24 h and 48 h; (**B**) lipids (area/average); (**C**) photomicrographs of *C. elegans* at 100× magnification. Results are expressed as means ± SEM. * *p* < 0.05 and **** *p* < 0.0001 versus Control.

AECa did not alter the viability of the nematode *C. elegans*, except at a concentration of 1000 µg/mL over 24 h, where viability remained above 85%. At 48 h, the highest concentrations of AECa (750 and 1000 µg/mL) kept the viability of the nematodes above 75% (Figure 4A).

AECa reduced the lipid content in *C. elegans* by approximately 20% and 24% at concentrations of 250 and 500 µg/mL, respectively, compared to the Control (Figure 4B,C).

## 3. Discussion

This study reports, for the first time, the ability of the aqueous extract of *C. adamantium* leaves to reduce lipid content in preadipocytes, and in the in vivo model *C. elegans*, the production of proinflammatory cytokines in these cells, besides its antioxidant activity in vitro. In a previous study, we found that the main phytochemical constituents of AECa are quinic acid and phenolic compounds such as myricetin and quercetin [16]. Phenolic compounds are widely described for their antioxidant potential, as they have a chemical structure consisting of an aromatic ring and free hydroxyls that confer on them the ability to donate hydrogen and electrons [17,18]. AECa showed antioxidant activity in the DPPH^•^, FRAP, and β-carotene bleaching assays, probably mediated by donating electrons from its phenolic compounds to stabilize radical molecules, reduce stable molecules, and prevent oxidative processes in lipids, respectively.

In addition, some phenolic compounds have been described as having anti-obesity potential, either through their ability to reduce adipogenesis and lipogenesis or to activate lipolysis [19,20,21]. In this study, we found that AECa reduced lipid accumulation when preadipocytes were treated with the extract simultaneously with the induction of differentiation with adipogenic medium. This reduction occurred in a concentration-independent manner of the AECa, which is probably related to the maximum sustained activity of the active compound in the extract, as observed for other plant extracts [22]. Probably, the AECa lowers adipogenesis (reduced differentiation) or reduces lipogenesis in differentiated cells, as some compounds present in AECa, such as quercetin and myricetin, have already been described as negative regulators of adipogenic and lipogenic genes and transcription factors such as PPAR-γ and SREBP-1c [23,24]. In this way, we can suggest that the effect of AECa is related to the group of compounds present in the extract that probably promote a reduction in the differentiation and lipogenesis of preadipocytes and adipocytes, respectively.

Additionally, AECa was able to promote lipolysis in differentiated and hypertrophied cells. Among the compounds in the extract, quercetin and myricetin may be associated with the lipolytic potential observed, as they have already been described in other studies for their lipolytic activity in adipocytes [23,25,26]. In a study by Yun-Soo et al. [25], quercetin promoted lipolysis by increasing the expression of hormone-sensitive lipase (HSL). In another study, Brasaemle et al. [26] found that myricetin downregulated the expression of perilipin A, the most abundant protein associated with lipid droplets in adipocytes that control basal and stimulated lipolysis [27].

The progression of obesity and its comorbidities is directly related to the increased production of proinflammatory cytokines and reduced production of anti-inflammatory cytokines [28]. In obesity, hypertrophied adipose tissue is accompanied by inadequate vascularization, making the cells dysfunctional and hypoxic and consequently increasing the production of proinflammatory cytokines, promoting immune cell recruitment [29]. Proinflammatory cytokines produced by immune cells and secreted by the adipose tissue, such as TNF-α, IL-4, and IL-6, stimulate obesity-associated diseases, including insulin resistance, dyslipidemia, and cardiovascular diseases. These comorbidities are related to the increased production of reactive oxygen species, which characterizes obesity-related oxidative stress [30,31,32,33]. Therefore, the in vitro antioxidant activity observed in this study may contribute to reducing the damage caused by obesity, as already observed in other studies [34].

AECa reduced the production of proinflammatory cytokines in the hypertrophied adipocytes in this study, which was probably mediated by the phenolic compounds and quinic acid present in AECa. In other studies, compounds present in AECa, such as phenolic compounds and quinic acid, have been shown to modulate cytokines such as TNF-α, IL-6, and MCP-1 [35,36,37]. Quinic acid, quercetin, and myricetin reduce TNF-α and IL-6 production levels by blocking the nuclear translocation of nuclear factor-κB [35,36]. Additionally, quercetin reduces the levels of MCP-1 in vitro and in vivo [37].

In the same way that AECa reduced lipid content in cells, it also reduced lipid accumulation in *C. elegans*, a complete organism. This free-living nematode is widely used as a model organism for evaluating the effect of different substances due to genes homologous to humans [38] and has been suggested as a model for obesity and drug studies [39]. *C. elegans* does not have adipocytes, so the lipid droplets are stored mainly in intestinal cells, called enterocytes. The lipid droplets of *C. elegans* mainly store triglycerides and cholesterol esters [40]. Lipid storage in *C. elegans* occurs through the activation of pathways orthologous to human pathways related to lipid metabolism, such as SBP-1, and those orthologous to SREBP [41].

In our study, *C. elegans* nematodes were treated with AECa from the first larval stage after the eggs had hatched. Therefore, the reduction in lipid content observed in the nematode intestinal cells is probably related to the suppression of the SBP-1 pathway by the compounds present in the extract. As previously mentioned, compounds present in AECa, such as myricetin, have been reported to modulate SREBP-1c negatively [42]. Moreover, another study observed that quercetin and myricetin reduced the lipid content in *C. elegans* [42].

Additionally, lipolysis in *C. elegans* can be mediated by hormone-sensitive lipase (HSL) [41]. As we saw earlier, among the compounds present in the extract, quercetin may be associated with the lipolytic potential observed by increasing the expression of HSL [23,25], thereby contributing to the reduction in the lipid content in *C. elegans* once the nematodes were treated until the L4 stage.

In conclusion, our results indicate that the aqueous extract of *C. adamantium* leaves has anti-obesity potential, with the ability to reduce adipogenesis and lipogenesis, activate lipolysis, and reduce the inflammatory processes and oxidative stress associated with the development and progression of obesity and its comorbidities.

## 4. Materials and Methods

### 4.1. Plant Material and Extract Preparation

*C. adamantium* O. Berg leaves were collected after authorization from SISBIO (Biodiversity Authorization and Information System, number 54470) in Dourados, Mato Grosso do Sul, Brazil (coordinates: 22°02′47.9″ S and 055°08′14.3″ W). An exsiccate was deposited in the Herbarium of the Federal University of Grande Dourados, Mato Grosso do Sul, Brazil (DDMS registration number 4108). The leaves were sanitized, dried in an air circulation oven at 45 °C, and pulverized in a knife mill. The aqueous extract of *C. adamantium* leaves (AECa) was prepared as described by Espindola et al. [15].

### 4.2. Evaluation of the Antioxidant Activity of AECa

#### 4.2.1. DPPH^•^ Free Radical Scavenging Assay

The antioxidant capacity of AECa was evaluated using the 2,2-Diphenyl-1-picrylhydrazyl (DPPH^•^) free radical capture method described by Bobo-García et al. [43]. Briefly, 20 µL of AECa (10–1000 µg/mL) was mixed with 180 µL of DPPH^•^ 150 µmol/L solution and incubated for 40 min at room temperature in the dark. The absorbance was measured at 515 nm. The Control consisted of 20 µL of distilled water and 180 µL of DPPH^•^ solution. Ascorbic acid and 2,3-tert-butyl-4-hydroxyanisole (BHA) (10–1000 µg/mL) were used as the standards. Three independent experiments were carried out in triplicate. The DPPH^•^ inhibition percentage was obtained using the following equation:

DPPH^•^ inhibition (%) = (1 − Abs_extract_/Abs_control_) × 100. (1)

The free radical 50% inhibition values (IC_50_) were calculated.

#### 4.2.2. Ferric Reducing Antioxidant Power (FRAP) Assay

The antioxidant capacity was assessed by its potential to reduce iron, as described by Ustundag et al. [44]. Briefly, 20 µL of AECa (20–200 µg/mL) was mixed with 280 µL of FRAP reagent. The mixture was incubated at 37 °C for 30 min. The absorbance was measured at 593 nm. The Control consisted of 20 µL of distilled water mixed with 280 µL of the FRAP reagent. BHA (20–200 µg/mL) was used as a standard. Three independent experiments were carried out in triplicate. The mean effective concentration (EC_50_) values of the extract were calculated.

#### 4.2.3. β-Carotene Bleaching Inhibitory Activity Assay

The antioxidant capacity was evaluated by inhibiting the bleaching of β-carotene, resulting from the oxidation induced by linoleic acid degradation products, as described by Koleva et al. [45] and Rocha et al. [46]. Briefly, 30 µL of AECa (100–1000 µg/mL) was mixed with 250 µL of β-carotene emulsion. The mixture was stirred and the absorbance was measured at 492 nm immediately after adding the emulsion (Abs1). The mixture was kept at 50 °C under stirring for 120 min. The absorbance was measured at 492 nm (Abs2). The Control consisted of 30 µL of distilled water and 250 µL of the emulsion. BHA was used as a standard (1–100 µg/mL). Three independent experiments were carried out in triplicate. The percentage of inhibition of β-carotene bleaching in the samples was obtained using the following equation:

Inhibition of β-carotene bleaching (%) = [1 − (Abs2_extract_ − Abs1_extract_)/(Abs2_control_ − Abs1_control_)] × 100. (2)

IC_50_ values were calculated.

### 4.3. Evaluation of the Effect of AECa on Preadipocytes and Adipocytes

#### 4.3.1. Cell Culture

Preadipocytes (3T3-F442A cells) were grown to 90% confluence in high-glucose Dulbecco’s Modified Medium (DMEM) containing 3.7 g/L of sodium bicarbonate and 5.77 g/L of HEPES, supplemented with 10% Fetal Bovine Serum (FBS) and 1% penicillin/streptomycin (10,000 U mL^−1^), all from Gibco/Invitrogen, Minneapolis, MN, USA. The cells were kept at 37 °C in a humidified atmosphere of 5% CO_2_.

#### 4.3.2. Cell Viability Assay Using MTT

Preadipocytes were plated in 96-well microplates (6 × 10^3^ cells/well) and incubated for 24 h. After this period, different concentrations of AECa (3.12–100 µg/mL) were added to the cell culture medium, and the cells were incubated for 24 h and 48 h. Subsequently, the cells were incubated for 4 h with 1 mg/mL of 3-(4,5-dimethylthiazol-2-yl)-2,5-diphenyltetrazolium bromide (MTT). The reduction in MTT in the mitochondria resulted in the formation of violet-colored formazan crystals, which were dissolved in DMSO (100 μL/well), and the absorbance was measured at 630 nm in a microplate reader. Three independent experiments were performed in triplicate.

#### 4.3.3. Lipid Accumulation in Cells

The effect of AECa on preadipocytes was evaluated according to the methods described by Elfakhani et al. [47] with modifications. For this purpose, preadipocytes were plated in 6-well plates (1 × 10^4^ cells/well). When 70% confluence was reached (day 0), the adipogenic medium was added for differentiation: cell culture medium, insulin 5 µg/mL, and rosiglitazone 3 mM.

To assess the antiadipogenic effect of AECa, the cells induced to differentiate with the adipogenic medium were simultaneously given different concentrations of AECa (25–100 µg/mL) on day 0 and incubated for 48 h. The adipogenic medium without AECa was replaced every 48 h until day 8. In the end, the cells were fixed with 10% formaldehyde for 1 h and stained with a 0.3% Oil red solution for 30 min. The cells were photographed in representative fields with a camera attached to an inverted microscope at 400× magnification for analysis. The stained lipid content was removed with 100% isopropanol, and the absorbance was measured at 492 nm.

To assess the lipolytic effect of AECa, hypertrophied cells were treated with different concentrations of AECa (25–100 µg/mL) on day 8 after differentiation. After 48 h of treatment, the cells were fixed with 10% formaldehyde for 1 h and stained with a 0.3% Oil red solution for 30 min. The stained lipid content was removed with 100% isopropanol, and the absorbance was measured at 492 nm.

The cells in the Control group were incubated with the cell culture medium without the adipogenic medium while untreated cells induced to differentiation were incubated with the adipogenic medium. Two independent experiments were performed in duplicate.

#### 4.3.4. Cytokine Quantification

To evaluate the effect of AECa on cytokine production (MCP-1, TNF-α, IL-6, and IL-10), the cells were treated with different concentrations of AECa (25–100 µg/mL) on day 0. On day 8, the adipocyte supernatant was collected and subjected to the Cytometric Bead Array (CBA) method (BD Biosciences). The evaluation was carried out by Flow Cytometry (BD LSRFortessa, BD biosciences) according to the manufacturer’s instructions, and analysis was performed using the (BD biosciences). Two independent experiments were performed in duplicate.

### 4.4. Evaluation of the Effect of AECa on Caenorhabditis elegans

#### 4.4.1. *C. elegans* Culture

The in vivo tests were carried out using the wild strain N2 of the nematode *C. elegans*, obtained from the Caenorhabditis Genetics Center (CGC), Minneapolis, MN, USA. The nematodes were incubated at 20 °C in Petri dishes containing nematode growth medium (NGM) and fed with *Escherichia coli* OP50 bacteria, inactivated with 10 mM kanamycin antibiotic. The nematodes were synchronized for the tests with an alkaline medium containing 2% sodium hypochlorite and 5 M sodium hydroxide. Eggs resistant to alkaline lysis were transferred to Petri dishes containing NGM and *E. coli*.

#### 4.4.2. Sub-Chronic Toxicity

The effect of AECa on the viability of *C. elegans* was evaluated according to the method described by Leite et al. [48]. Briefly, the nematodes were synchronized at the L4 stage of development (10 nematodes N2/well) and transferred to 96-well microplates containing 100 μL of M9 buffer and 100 μL of AECa at different concentrations (3.1–100 μg/mL). The nematodes were incubated at 20 °C for 24 h and 48 h. The nematodes in the Control group were incubated with M9 buffer (200 μL). In the end, nematode viability was assessed by touch sensitivity using a platinum wire. Three independent experiments were carried out in triplicate.

#### 4.4.3. Lipid Accumulation in *C. elegans*

For the quantification of lipids in *C. elegans*, the method described by Escorcia et al. [49] was used. For this purpose, synchronized *C. elegans* nematodes (L1 stage) were transferred to Petri dishes containing NGM and 300 μL of different concentrations of AECa (250–500 μg/mL) diluted in *E. coli* OP50. Upon reaching the L4 stage of development (approximately 48 h after synchronization), the nematodes were stained with 0.3% Oil red for 2 h. In the end, a slide was prepared with a volume of 5 µL, covered with a coverslip, and photographed under a microscope at 100× magnification. The images were analyzed using the ImageJ (version 1.52a) program. Three independent experiments were performed in duplicate.

### 4.5. Statistical Analysis

The data obtained were expressed as means ± standard error of the mean (SEM). Analysis of variance (ANOVA) with a Newman–Keuls post-test in GraphPad Prism 9 software (San Diego, CA, USA) was used to analyze and compare the experimental groups. The results were considered significant when *p* < 0.05.

## Figures and Tables

**Table 1:** Antioxidant activity of aqueous extract of *Campomanesia adamantium* leaves (AECa).

Sample	DPPH^•^	FRAP	β-Carotene Bleaching
	IC_50_ (µg/mL)	EC_50_ (µg/mL)	IC_50_ (µg/mL)
AECa	10.41 ± 1.06	56.54 ± 3.00	229.87 ± 5.92
AA	6.09 ± 2.28	40.16 ± 4.96	ND
BHA	6.45 ± 2.28	-	15.14 ± 2.07

AA: ascorbic acid; BHA: butyl-4-hydroxyanisole; IC_50_: half-maximal inhibitory concentration; EC_50_: median effective concentration; -: not performed; ND: not detected. Results are expressed as means ± SEM.

## Data Availability

The data presented in this study are available upon request from the corresponding author.
